# Response to the commentary on “Pre- and post-COVID-19 pandemic identification of dengue hotspots and exploration of determinants in Quezon City, Philippines”

**DOI:** 10.1186/s41182-025-00858-7

**Published:** 2026-01-21

**Authors:** John Robert Carabeo Medina, Shin’ya Kawamura, Rie Takeuchi, Rolando V. Cruz, Johnedel Mendoza, Paul Michael R. Hernandez, Fernando B. Garcia, Ernesto R. Gregorio, Jun Kobayashi

**Affiliations:** 1 Institute of Clinical Epidemiology, National Institutes of Health, University of the Philippines Manila, 623 Pedro Gil St, Ermita, 1000 Manila, Metro Manila Philippines; 2 Department of Clinical Epidemiology, College of Medicine, University of the Philippines Manila, 547 Pedro Gil St, Ermita, 1000 Manila, Metro Manila Philippines; 3 National Institute of Technology, Matsue College, 14-4 Nishi-Ikumacho, Matsue, Shimane 690-8518 Japan; 4 Graduate School of Public Health, International University of Health and Welfare, 4-3, Kodunomori, Narita, Chiba 286-8686 Japan; 5 Quezon City Epidemiology and Surveillance Division, Quezon City Health Department, Local Government of Quezon City, Quezon City, Philippines; 6 Department of Environmental and Occupational Health, College of Public Health, University of the Philippines Manila, 625 Pedro Gil St, Ermita, 1000 Manila, Metro Manila Philippines; 7 Healthy University Office, University of the Philippines Manila, Manila, Philippines; 8 Department of Health Policy and Administration, College of Public Health, University of the Philippines Manila, 625 Pedro Gil St, Ermita, 1000 Manila, Metro Manila Philippines; 9 Department of Health Promotion and Education, College of Public Health, University of the Philippines Manila, 625 Pedro Gil St, Ermita, 1000 Manila, Metro Manila Philippines; 10 Department of Global Health, Graduate School of Health Sciences, University of the Ryukyus, 207 Uehara, Nakagami-Gun, Nishihara-Cho, Okinawa 903-0215 Japan

Dear Editor,

We appreciate the authors of the recent commentary [1] for their thoughtful engagement with our study [2], which we believe has contributed to advancing understanding of dengue dynamics in urban settings through collegial discussions. Their reflections emphasize the importance of continued dialogue in epidemiologic research, and we welcome this opportunity to clarify our study’s aims and methodological context.

## On theoretical frameworks and ecological design

Theoretical framework is vital for it informs the conceptual framework, which is the operational blueprint of an epidemiologic study. Our study was explicitly framed as exploratory and data-driven, designed to identify spatial patterns and determine ecological associations. While we did not explicitly state that the analysis is following a singular theoretical model, it is apparent that the conceptual model is influenced by the ecologic model aiming to show how urbanization, transportation, and greenness may relate to the occurrence of dengue in the city. The representative variables for those concepts have already been investigated and were found to be contributing to dengue transmission dynamics.

We would also like to clarify the confusion on the descriptive nature versus the explanatory nature of the study. While we stated that the study design is an explanatory, multi-group comparison ecologic design, we did not mean to say that this falls under the realm of analytic epidemiology as our study remains an ecologic study. According to Susser [3], ecological studies may be exploratory (descriptive) or analytical. Furthermore, ecologic designs may be classified on two dimensions, which are the method of exposure measurement and method of grouping. Ecological studies can be exploratory if there is no exposure of interest to be included in the analysis, or explanatory (etiologic) if an ecological association between the summary measures of the exposure and outcomes of the different groups will be explored. Groups of an ecological study can be differentiated by place (multi-group comparison design), by time (time-trend design), and by a combination of the two (mixed design) [4]. Since we tested ecological associations between urban determinants and dengue incidence across spatial units, the study design was an explanatory, multi-group comparison ecologic design. This reflects a move beyond description into explanatory analysis, but remains within the bounds of ecological inference. We sought to understand why certain areas exhibit persistent or shifting hotspots, not merely where they occur.

## On confounding variables and biases

The commentary raises valid concerns about confounding, which we addressed by being transparent about it in the limitations of the study. It is important to note that ecological designs inherently operate at the population level, and the ability to control for confounding is constrained by the absence of individual-level data. While we conducted crude analyses, this is consistent with the nature of ecological studies, which are often hypothesis-generating and descriptive by design. We deliberately avoided causal language and flagged the risks of selection bias and ecological fallacy. As Morgenstern [5] notes, ecological fallacy arises when group-level associations are misinterpreted as individual-level effects. It was a misstep that we explicitly avoided.

Regarding potential reporting bias, we acknowledged in the limitations of our study that dengue surveillance might have been compromised during the height of the COVID-19 pandemic, potentially affecting observed trends. However, the local surveillance system in Quezon City remained operational throughout this period. Each barangay maintained a functioning health center that continued to address residents’ health needs. Moving forward, further observational studies can be conducted in the identified dengue hotspots, ideally incorporating individual-level analyses to better understand transmission dynamics and risk factors.

## On data triangulation

We agree that triangulation enhances robustness. However, our study’s design involved spatial analysis using official surveillance and population data, as well as satellite images of buildings and facilities, greenness and surrounding greenness, and pick-up land use from Sentinel-2 and OpenStreetMap. These data sources and methods were clearly described in our methods. We did not claim to offer a mixed-methods approach. We welcome future studies that incorporate entomological, qualitative, and molecular data to enrich the understanding of dengue transmission dynamics at the individual level. However, we are also aware that such integration may require more substantial operational costs and logistical support. In countries burdened with several diseases, research on neglected tropical diseases including dengue is sometimes sidelined, if not often, as resources and research funds are allocated more to urgent or priority public health threats that require immediate attention [6]. Nevertheless, advancing dengue research should ensue despite the challenges in funding availability.

## On policy translation

While we did not map a full research-to-policy pathway, we emphasized the relevance of our findings for local-level planning and resource allocation. We appreciate the call for stronger operational linkages and agree that future research should prioritize co-production with public health stakeholders. We are also pleased to share that our research team is actively collaborating with the Quezon City Epidemiology and Surveillance Division (QCESD), which has already adopted hotspot analysis in their routine surveillance practices. This uptake reflects the practical value of our findings and signals a growing synergy between academic research and local public health action. Although not all operational developments could be captured within the scope of the published article, meaningful work continues on the ground that bridges data with decision-making and translates evidence into implementation. This ongoing collaboration has inspired us to pursue a dedicated paper that documents this translational process and its implications for local health systems strengthening.

## On literature coverage

The commentary suggests that our review omitted recent evidence on vectorial capacity and molecular data. We focused our synthesis on spatial and ecological determinants relevant to our study design. Nonetheless, we acknowledge that broader literature integration could strengthen contextual interpretation, and we welcome this reminder for future work.

In closing, we thank the authors for their constructive critique. Their commentary reinforces the need for methodological rigor, theoretical clarity, and translational relevance in dengue research. We remain committed to improving the quality and utility of our work and hope that this exchange contributes to a more nuanced and collaborative scientific dialogue.

We also respectfully urge readers to appreciate the unique strengths of ecological study designs. These are not lesser versions of cross-sectional studies, case–control studies, cohort studies or experimental studies. Ecologic designs are distinct approaches tailored to population-level questions. Their beauty lies in capturing community-level dynamics, revealing structural determinants, and guiding interventions at scale. Especially in urban health and infectious disease surveillance, ecological designs offer critical insights that are both practical and policy-relevant. However, caution must always be observed in interpreting findings and drawing conclusions, especially to avoid ecological fallacy, where group-level associations are mistakenly inferred as individual-level effects. We hope that future critiques will engage with ecological studies on their own terms, recognizing their methodological integrity and real-world utility.

## Data Availability

Not applicable.
